# Supplementary material for: Gut microbiota diversity in human strongyloidiasis differs little in two different regions in endemic areas of Thailand
Source: PLoS One. 2022 Dec 30;17(12):e0279766. doi: 10.1371/journal.pone.0279766 (PMC9803247; doi:10.1371/journal.pone.0279766)
Supplement: S1 Fig — (PDF) [file pone.0279766.s001.pdf]

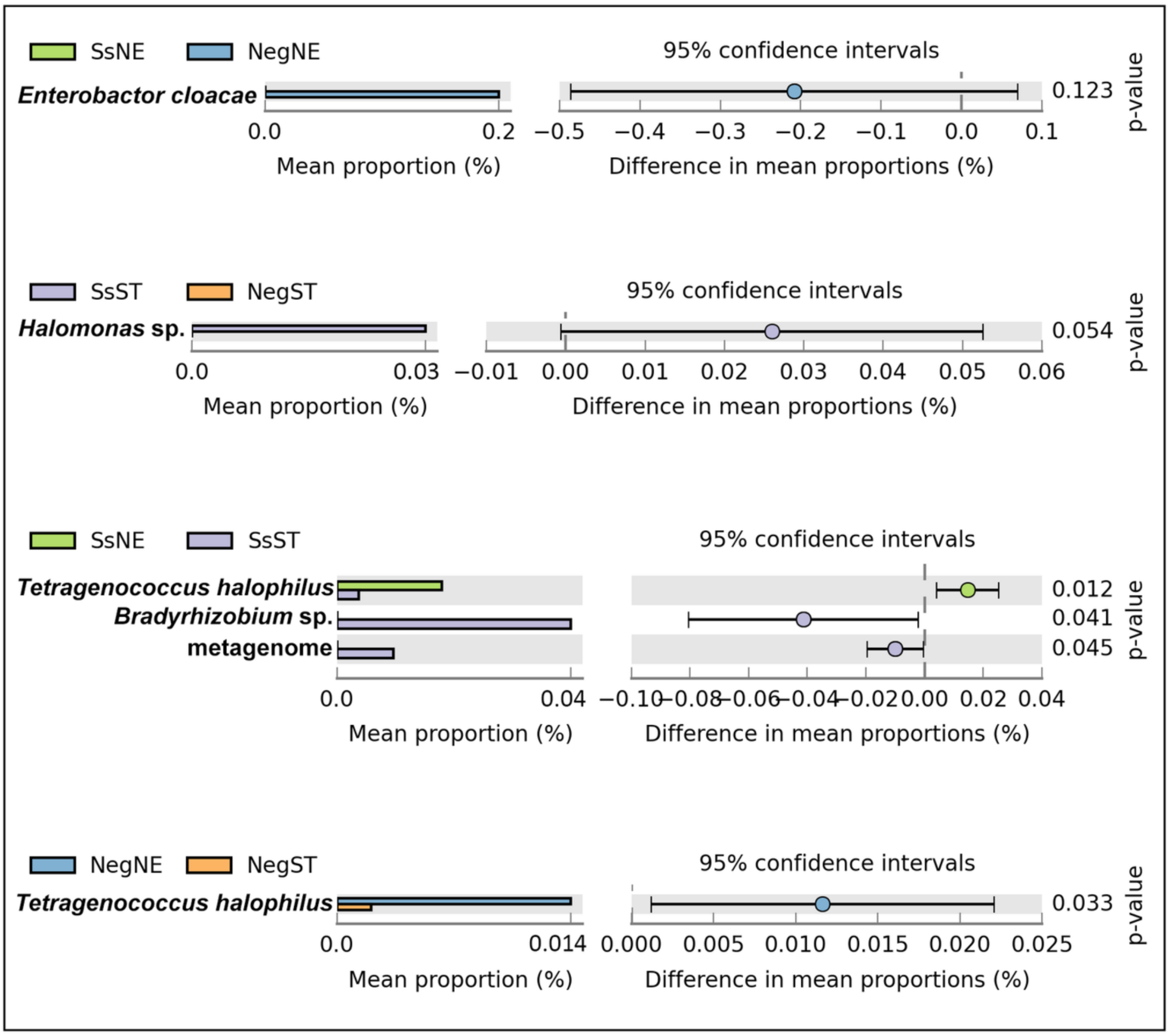

S1 Fig. Between-group variation analysis of selected species using t-tests.

The left panel shows species with marked between-group differences in representation. Each bar represents the mean value of the species abundance. The right panel indicates the mean (colored circle) and confidence interval (upper and lower 95% limits) for between-group comparisons. The color of each circle indicates the group whose mean value is higher. The right-most value is the p-value of the significance test of between-group variation.
