# Supplementary material for: Gut microbiota diversity in human strongyloidiasis differs little in two different regions in endemic areas of Thailand
Source: PLoS One. 2022 Dec 30;17(12):e0279766. doi: 10.1371/journal.pone.0279766 (PMC9803247; doi:10.1371/journal.pone.0279766)
Supplement: Attachment — Submitted filename: Review-update.docx [file pone.0279766.s006.docx]

The manuscript entitled “Gut microbiota diversity in patients with strongyloidiasis differs little in two different regions in endemic areas of Thailand” by Rodpai et al, showed that *S. stercoralis* infection leads to only minor alterations in the relative abundance of individual bacterial species in the human gut, and no detectable effect was observed on community structure and diversity. Some differences in species abundance were noted between geographical isolates. There are several points concerning clarity in the manuscript.

- line 72-74: S*. stercoralis* infection prevalence in 4 regions of Thailand. Explain more why the study area focused only in the southern and the northeastern areas and only one province from each area was selected.

- Please give more information about the sample size. Only 10 stool samples (5 SS positive and 5 SS negative) from each province were used for microbial composition analysis.

- line 85- amount of fecal samples and number of volunteers should be added

- Diet, age and health condition play an important role on microbial gut composition, therefore basic information of the volunteers, for examples age, health condition, diet, should be added.

-line 89- A portion means how many grams of each fecal sample

-line 91 The stool samples were categorized in 4 groups. Give more detail about DNA preparation of stool samples from each area. Is it prepared separately, or are pool samples from each area together and then extracted for the DNA?

Discussion

Line 243- please discuss more about the Ruminococcaceae abundance in uninfected groups and low abundance in infected groups.
